# Inoculation with *Bacillus megaterium* CNPMS B119 and *Bacillus subtilis* CNPMS B2084 improve P-acquisition and maize yield in Brazil

**DOI:** 10.3389/fmicb.2024.1426166

**Published:** 2024-06-26

**Authors:** Christiane Abreu de Oliveira-Paiva, Daniel Bini, Sylvia Morais de Sousa, Vitória Palhares Ribeiro, Flávia Cristina dos Santos, Ubiraci Gomes de Paula Lana, Fabiane Ferreira de Souza, Eliane Aparecida Gomes, Ivanildo Evódio Marriel

**Affiliations:** ^1^Microbiology Laboratory, Embrapa Milho e Sorgo, Sete Lagoas, Brazil; ^2^Applied Biology Center, Embrapa Milho e Sorgo, Sete Lagoas, Brazil

**Keywords:** Bacillaceae, bacteria, co-inoculation, maize yield, phosphate-solubilizing, plant growth promoting bacteria

## Abstract

Phosphorus (P) is a critical nutrient for plant growth, yet its uptake is often hindered by soil factors like clay minerals and metal oxides such as aluminum (Al), iron (Fe), and calcium (Ca), which bind P and limit its availability. Phosphate-solubilizing bacteria (PSB) have the unique ability to convert insoluble P into a soluble form, thereby fostering plant growth. This study aimed to assess the efficacy of inoculation of *Bacillus megaterium* B119 (rhizospheric) and *B. subtilis* B2084 (endophytic) via seed treatment in enhancing maize yield, grain P content, and enzyme activities across two distinct soil types in field conditions. Additionally, we investigated various mechanisms contributing to plant growth promotion, compatibility with commercial inoculants, and the maize root adhesion profile of these strains. During five crop seasons in two experimental areas in Brazil, Sete Lagoas-MG and Santo Antônio de Goiás-GO, single inoculations with either B119 or B2084 were implemented in three seasons, while a co-inoculation with both strains was applied in two seasons. All treatments received P fertilizer according to plot recommendations, except for control. Both the *Bacillus* strains exhibited plant growth-promoting properties relevant to P dynamics, including phosphate solubilization and mineralization, production of indole-3-acetic acid (IAA)-like molecules, siderophores, exopolysaccharides (EPS), biofilms, and phosphatases, with no antagonism observed with *Azospirillum* and *Bradyrizhobium*. Strain B2084 displayed superior maize root adhesion compared to B119. In field trials, single inoculations with either B119 or B2084 resulted in increased maize grain yield, with relative average productivities of 22 and 16% in Sete Lagoas and 6 and 3% in Santo Antônio de Goiás, respectively. Co-inoculation proved more effective, with an average yield increase of 24% in Sete Lagoas and 11% in Santo Antônio de Goiás compared to the non-inoculated control. Across all seasons, accumulated grain P content correlated with yield, and soil P availability in the rhizosphere increased after co-inoculation in Santo Antônio de Goiás. These findings complement previous research efforts and have led to the validation and registration of the first Brazilian inoculant formulated with *Bacillus* strains for maize, effectively enhancing and P grain content.

## 1 Introduction

Phosphorus (P) ranks as the second most crucial nutrient for plants, right after nitrogen (N). Various plant processes rely on P, including photosynthesis, respiration, energy production, and the biosynthesis of nucleic acids (Vance et al., 2003). In many tropical agricultural soils, the total P content can be notably high (200–800 mg kg^−1^), yet only a small portion of it is readily available due to its high sorption capacity (Pavinato and Rosolem, 2008; Alori et al., 2017). This phenomenon occurs because soluble P tends to strongly bind to clay minerals and metal cations like calcium (Ca), iron (Fe), and aluminum (Al) (Hinsinger, 2001; Kalayu, 2019; Pavinato et al., 2020), rendering it inaccessible for root absorption.

To address this challenge, agricultural crops have been supplemented with high doses of P fertilizers to enhance productivity. However, it is estimated that approximately 70–80% of the P applied does not become available to plants due to soil sorption mechanisms that restrict its mobility (Hinsinger, 2001; Khan et al., 2007; Pavinato et al., 2020). This practice presents economic and environmental challenges as P-fertilizer is predominantly imported and sourced from non-renewable resources. Moreover, fertilizer prices have escalated due to conflicts in Eastern Europe, a key producer of these inputs, highlighting the vulnerability of the global production system reliant on external fertilizer markets in this region. Brazil, renowned as a major grain producer and exporter, heavily relies on imported fertilizers. It ranks as the fourth-largest importer of N, P and potassium (K) globally, importing around 95% of K, 60% of P, and 80% of N (Agrolink, 2023). Challenges related to the use of P-fertilizers and shifts in the global market have prompted the agricultural sector to explore new alternatives aimed at reducing production costs and adopting more sustainable agricultural practices. In this context, there is been increasing encouragement for the use of microbial inoculants, emerging as a significant agricultural technology capable of modernizing practices, cutting costs, and aligning with the Sustainable Development Goals outlined by the United Nations.

Microbial inoculants are primarily composed of plant growth-promoting rhizobacteria (PGPR), offering numerous mechanisms that benefit plants (Javed et al., 2021; O’Callaghan et al., 2022). These mechanisms include biological nitrogen fixation, phosphorus solubilization and mineralization, biological control of pests and diseases, protection against water and nutrient stresses, stimulation of root growth, among others (Patil et al., 2012; O’Callaghan et al., 2022; Silva et al., 2023). The search for phosphate-solubilizing bacteria (PSB) remains a common focus in the study of soils exhibiting high P-sorption capacity. Over the years, significant efforts have been dedicated to assessing the potential of bacterial strains in phosphate solubilization to enhance crop yield. Several PGPRs are recognized as PSBs, including *Pseudomonas, Burkholderia, Bacillus, Bradyrhizobium, Rhizobium, Gluconacetobacter, Herbaspirillum,* and *Azospirillum* (Tabassum et al., 2017; Elhaissoufi et al., 2022; Silva et al., 2023). Among these, the *Bacillus* genus has received considerable attention and stands out as a well-studied group with longstanding significance in agriculture and various other fields (Aloo et al., 2018; Biedendieck et al., 2021; Sharf et al., 2021).

The *Bacillus* genus encompasses approximately 400 species exhibiting diverse physiological, metabolic, and phenotypic characteristics (Parte et al., 2020). It represents a heterogeneous group of bacteria found across different environments, characterized by rod-shaped cells, the formation of endospores, aerobic or facultative anaerobic activity, and catalase positivity (Dworkin et al., 2006; Setiawati et al., 2022). Historically, *Bacillus megaterium* var. *phosphaticum* (now renamed *Priestia megaterium* – Gupta et al., 2020; Biedendieck et al., 2021) was the first bacterium utilized as a P inoculant in the Soviet Union in 1950, marketed under the name Fosfobacterin (Tisdale and Nelson, 1975; Khan et al., 2007; Setiawati et al., 2022). Numerous studies have demonstrated that *B. megaterium*, alongside other *Bacillus* species, exhibits a remarkable ability to colonize the rhizosphere of grasses, solubilize phosphate (via organic and inorganic acid release and proton extrusion), mineralize phosphate (via phosphatase production), and produce phytohormones and siderophores (Velloso et al., 2020; Silva et al., 2023). Additionally, their capacity to form endospores enhances their adaptation to various abiotic conditions such as extreme temperatures, pH levels, or radiation exposure (Biedendieck et al., 2021).

Various P inoculants containing *Bacillus* and other PSBs are available on the global market, manufactured in Asia and Europe through the selection of regional strains. Examples include Jumpstar (O’Callaghan et al., 2022), Bio-Phospho, Bio Promotor Phosphobacteria, Potash solubilizing liquid, and Biozote-P (Tabassum et al., 2017). Many of these inoculants comprise a blend of phosphate solubilizers, rock phosphate, and a carbon source (e.g., sugarcane or cassava) and are distributed in countries such as Canada, Australia, Egypt, and India. In Brazil, commercial inoculants formulated with native PSBs were not available until 2019 when the BiomaPhos inoculant was introduced to the market. This development followed preliminary *in vitro* tests, greenhouse, and field evaluations of *B. megaterium* CNPMS B119 and *B. subtilis* CNPMS B2084 strains for maize inoculation, demonstrating growth promotion and phosphate solubilization mechanisms (Oliveira et al., 2009; Gomes et al., 2014; Ribeiro et al., 2018; Velloso et al., 2020; Sousa et al., 2021; Santos et al., 2022). Maize holds a significant position in the world, including Brazilian agricultural sector, underlining the importance of seeking alternative practices and technologies to enhance its productivity while minimizing costs and reducing reliance on external fertilizers, thereby benefiting the environment. Therefore, this study focused on two aims with *B. megaterium* CNPMS B119 and *B. subtilis* CNPMS B2084 strains: (i) evaluating mechanisms for promoting plant growth, root adhesion, and compatibility with strains of commercial inoculants and (ii) assessing and presenting the outcomes of grain yield and P acquisition in maize inoculated during five crop seasons in two experimental areas in Brazil. These experiments played a pivotal role in the development and commercialization of the first inoculant formulated with PSB specifically for maize in Brazil (Registration number: PR 000497-9.000045).

## 2 Materials and methods

### 2.1 *Bacillus* strains

The *Bacillus* strains, *B. megaterium* CNPMS B119 (referred to as B119) and *B. subtilis* CNPMS B2084 (referred to as B2084), are part of the Collection of Multifunctional Microorganisms and Phytopathogens (CMMF) of Embrapa Maize and Sorghum. Strain B119 was isolated from rhizosphere and strain B2084 from the leaf endosphere of P-efficient tropical maize genotypes (Oliveira et al., 2009; Abreu et al., 2017). Moreover, they were selected through agronomic screenings involving maize, millet, and sorghum under controlled conditions (Ribeiro et al., 2018; Velloso et al., 2020), as well as field trials with other PSM strains (Sousa et al., 2021).

This study consisted of two phases. First, *in vitro* tests were conducted to identify additional properties that complement the PGPR capabilities of *B. megaterium* B119 and *B. subtilis* B2084. Second, agronomic field trials were conducted with maize crops to evaluate the effects of single and co-inoculation of *Bacillus* strains.

### 2.2 *In vitro* experiments

#### 2.2.1 Osmotic stress tolerance

Osmotic stress tolerance was determined using the method described by Velloso et al. (2020). *Bacillus* strains were inoculated in a culture medium consisting of 10% (w/v) Tryptone Soy Agar (TSA) supplemented with sorbitol at a concentration of 405 g L^−1^. The cultures were then incubated at 30°C for 72 h. This medium creates an environment with reduced water activity, corresponding to a value (Aw) of 0.919.

#### 2.2.2 Exopolysaccharides (EPS) and biofilm productions

The assessment of EPS production followed the protocol outlined by Paulo et al. (2012). This evaluation relied on observing the formation of a mucoid colony around the discs, which was confirmed by mixing a portion of the mucoid substance in 2 mL of absolute ethanol. A positive result was indicated by the formation of a precipitate, while a negative result was indicated by turbidity. Biofilm production was determined utilizing a spectrophotometric method as described by Stepanović et al. (2007).

#### 2.2.3 Siderophore and phytase productions

Quantitative siderophore production was conducted following the method proposed by Arora and Verma (2017), with a modification in the incubation time extended to 120 h. Extracellular and intracellular phytase productions were determined according to the method described by Greiner (2007) and detailed by Velloso et al. (2020).

#### 2.2.4 Acid and alkaline phosphatase productions

*Bacillus* (10^8^ CFU mL^−1^) was inoculated in triplicate into National Botanical Research Institute’s phosphate growth medium (NBRIP) (Nautiyal, 1999) adjusted to pH 7 followed by incubation at 30°C and 150 rpm. *Pseudomonas aeruginosa* strain BRM 046308 from CMMF served as the positive control. After 24, 48, 72, and 96 h of incubation, the samples were centrifuged at 3,350×*g* for 15 min. Subsequently, 150 μL of the supernatant were processed according to Tabatabai (1994) and measured on a spectrophotometer UV/VIS (Perkin Elmer, United States) at 400 nm. A standard curve was prepared using a p-nitrophenol (pNP) solution with concentrations ranging from 0 to 25 μg mL^−1^.

#### 2.2.5 Total acid organic production

The *Bacillus* strains were incubated in liquid medium containing either iron or tricalcium phosphate as a source of phosphorus, following the method described by Nahas et al. (1994). After ten days of incubation, aliquots of the liquid medium were collected to quantify organic acids using high-performance liquid chromatography (HPLC) equipment (Sigma-Aldrich, USA) in the Shimadzu Prominence Model LC-20A (Shimadzu, Japan). The separation of acids occurred at 65°C with 5 mM H_2_SO_4_ serving as the mobile phase, at a flow rate of 0.6 mL min^−1^. The Shimadzu RID-10A differential refractive index detector, with the cell temperature set at 45°C, was utilized to measure the concentration of organic acids. The area of the peak wavelength separation generated by the refractive index was calculated using standard curve.

#### 2.2.6 Phytate mineralization

Pure colonies of strains B119 and B2084 were cultivated in Trypticase Soy Broth (TSB) liquid medium for five days at 120 rpm and 28°C. Aliquots of 100 μL (10^8^ CFU mL^−1^) of each cell suspension were then transferred to a conical tube containing 20 mL of modified NBRIP supplemented with 40 g L^−1^ of glucose and 1 g L^−1^ of sodium phytate (inositol hexa- and pentaphosphate) as a source of phosphorus. After incubating for nine days at 120 rpm and 28°C, the cultures were centrifuged, and the supernatant was filtered through Whatman filter paper no. 42 to determine the concentration of soluble phosphorus. Readings were taken on a spectrophotometer UV/VIS (Perkin Elmer, United States) at 880 nm, following the method described by Murphy and Riley (1962).

#### 2.2.7 Biological N fixation

The experiment followed Döbereiner’s (1989) method, utilizing a nitrogen-free culture medium (NFb) to cultivate bacterial strains. In triplicate, tubes containing 3 mL of semisolid NFb medium were inoculated with 10 μL of bacterial culture per mL (10^8^ CFU mL^−1^). After incubating for five days at 30°C, bacteria that formed a visible growth film beneath the surface of the medium, causing a color change from green to blue, were identified as potential nitrogen fixers. As a positive control, the bacterium *Azospirillum brasilense* strain 1,626 from CMMF was utilized.

#### 2.2.8 Compatibility tests between bacterial strains

The cross-streak method, adapted from Lertcanawanichakul and Sawangnop (2008), was utilized to conduct the compatibility test. *Bacillus* strains (B119 and B2084) were tested for compatibility with the primary commercial strains commonly used in Brazilian agriculture, including *Bradyrhizobium diazoefficiens* (Semia 5080), *B. elkanii* (Semia 5019 and Semia 587), *B. japonicum* (Semia 5079), *Azospirillum* sp. (AbV5), and *Azospirillum* sp. (AbV6). Nutrient agar medium was employed for compatibility tests between *Bacillus* and *Azospirillum* strains, while Yeast Mannitol Agar (YMA) medium was used for tests between *Bacillus* and *Bradyrhizobium* strains.

Initially, each strain was grown separately in a specific medium (nutrient agar or YMA) at 29°C for three days. Subsequently, two strain combinations were tested for their compatibility by cross streak assay. For this, one strain was streaked vertically and the other horizontally in each plate, incubated at 29°C for three days. Incompatibility between bacterial strains was indicated by the presence of an inhibition zone in the intersection of the paired strains. Conversely, compatible strains showed no inhibition zone. A positive control for incompatibility was performed between *Pseudomonas aeruginosa* IPR 45 and *Bacillus velezensis* LIS05 strains from CMMF.

#### 2.2.9 Adherence of bacteria to root surface

The root adhering process of B119 and B2084 strains was performed as reported by Hozore and Alexander (1991). Root fragments of maize seedlings were aseptically immersed in the bacterial suspension containing 10^7^ cells mL^−1^ for 20 min. The number of viable cells was evaluated by counting their weak or strong adherence to the root. After contact with bacterial suspension, weak bacterial adhesion was determined by immersion of individual root fragments in 10 mL of saline solution (0.85% NaCl) for 15 s. After, each fragment was immersed again in 10 mL of saline solution and agitated at 150 rpm for 15 min. Serial dilutions were prepared (10^−1^ to 10^−6^) and plated in a nutrient agar medium. Count was performed after 48 h, and the results were registered in percentage considering the number of viable bacteria quantified in the initial inoculation solution.

### 2.3 Sample roots preparation for scanning electron microscopy analysis

Initially, preparation of microbial inoculants and growing maize seedlings by hydroponic method were performed as described by Sousa et al. (2021). Roots of maize seedling were soaking in bacteria solution containing 10^7^ UFC mL^−1^ by 6 and 72 h for each treatment. The treatments used were B119, B2084, B119 + B2084 and non-inoculated control [0.85% (w/v) NaCl]. Thick roots and fine roots were collected in each time post inoculation (6 h and 72 h) and fixed in Karnovsky solution (Karnovsky, 1965). The root samples were stored at 4°C for 24 h. Next, the roots were transferred to glycerin for 30 min before cutting in liquid nitrogen, followed by dehydration in acetone 25, 50, 75, 90 and 100% (v/v), being one wash for 10 min for each concentration up to 90% and three washes in 100% acetone. The samples were dried in a critical point[CPD 030 − (Bal − Tec)], fixed in the stub using double carbon tape, metalized in a gold evaporator [SCD 050 − (Bal − Tec)] and the observations were done in a Scanning Electron Microscope – SEM – FEG ultra-high resolution, field-free, CLARA model 2021 (TESCAN, Czech Republic).

### 2.4 Single and co-inoculation field experiments

#### 2.4.1 Experimental areas

The field experiments were conducted across two experimental areas, spanning five harvesting seasons, between the months of November and March in the years 2015, 2016, 2017, 2020, and 2021. We refer to each harvest season as a “season” or “crop season.” These areas were situated in Embrapa Research Stations located in the cities of Sete Lagoas, MG, Brazil (19°28’S 44°15’W; altitude 761 m), and Santo Antônio de Goiás, GO, Brazil (16°28’S 49°17’W; altitude 766 m).

The climate of Sete Lagoas is classified as Cwa under the Köppen system, characterized by high altitude tropical conditions with mild winters and hot, rainy, and humid summers. The dry months typically occur between April and September, with average temperatures of 21°C and 4 mm of rainfall, while the warmest and rainiest months are from October to March, with average temperatures of 25°C and 237 mm of rainfall. In contrast, the climate of Santo Antônio de Goiás is classified as Aw (tropical savannah, megathermal). Rainfall typically occurs from May to September, with average temperatures of 24°C and 194 mm of rainfall, while dry months extend from October to April, with average temperatures of 22°C and 21 mm of rainfall.

Our field experiments were performed according to Brazilian legislation (normative instruction no 53/2013, MAPA) in two representative regions of maize crops, with different soil types. The soil in Sete Lagoas is classified as a typical dystrophic Red Oxisol (Soil Taxonomy), Cerrado phase, characterized by a clayey texture with low phosphorus availability. Conversely, the soil in Santo Antônio de Goiás is classified as a Dark Red Oxisol (Soil Taxonomy), Cerradão phase, featuring a clayey texture and high fertility due to the residual effects of successive fertilization, often referred to as built fertility. Soil chemical characteristics are detailed in Table 1. The experimental irrigation was carried out using the sprinkle method, guided by soil water balance and utilizing crop evapotranspiration measurements obtained from a class A tank as an indicator of water consumption (Albuquerque, 2007).

**Table 1:** Soil chemical analysis at experimental areas in Brazil.

Parameters	Sete Lagoas	Santo Antônio de Goiás
pH_H2O_	5.8	6.0
P_Mehlich-1_ (mg dm^−3^)	3.9	15.9
K (mg dm^−3^)	20.0	257.3
Ca (cmol_c_ dm^−3^)	2.11	3.89
Mg (cmol_c_ dm^−3^)	0.57	1.67
Al (cmol_c_ dm^−3^)	0.02	0.01
H + Al^*^ (cmol_c_ dm^−3^)	9.07	9.68
SB^**^ (cmol_c_ dm^−3^)	2.74	6.21
V^***^ (%)	23.3	64.3
OM^****^ (g kg^−1^)	39.1	34.2

*H + Al: potential acidity; **SB: sum of bases; ***V: base saturation; ****OM: organic matter.

#### 2.4.2 Inoculant preparation

The inoculants were prepared using pure cultures of B119 and B2084. The strains were cultured in TSB medium for three days at 28°C with agitation at 120 rpm. Following this, the strain cultures were centrifuged for 10 min at 6,000×*g*, and the resulting pellets were resuspended in saline solution [0.85% (w/v) NaCl]. The cell density was adjusted to approximately 10^9^ cells mL^−1^ using spectrophotometer UV/VIS (Perkin Elmer, USA), at 540 nm. Subsequently, the adjusted suspension of each strain was mixed with a carrier, ground activated charcoal, at a proportion of 30% (w/v) of the total liquid. The resulting inoculant (bacterium + charcoal) was then added to the seeds after coating them with a 4% (w/w) cassava starch gum adhesive. The coated seeds were left to dry in the shade for 30 min before being used for sowing.

#### 2.4.3 Experimental designs

The single inoculation was carried out in field trials included three seasons of maize in Brazil (2015, 2016 and 2017), using a randomized block experimental design, with four replicates and four treatments. Each subplot consisted of four lines of 5 m with 0.70 m between rows. The treatments were the following: non-inoculation and non-phosphate-fertilization (control); no inoculation (B0); single inoculations with *B. megaterium* B119 and single inoculation of *B. subtilis* B2084. Treatments B0, B119 and B2084 were fertilized with triple superphosphate (TSP), according to the total dose recommended for maize crops (100 kg ha^−1^ of P_2_O_5_). For all treatments, the side-dress fertilization was divided into two applications of 150 kg ha^−1^ of urea at 30 and 45 days after sowing. The other cultural treatments were carried out according to regional recommendations. The experiment was carried out until the end of the maize growing period (around 120 days).

The co-inoculation was carried out following the agronomic validation of single inoculation, a field experiment was set up for two seasons to assess the efficacy of *Bacillus* co-inoculation. In this phase, a commercially formulated inoculant containing both B119 and B2084 strains (provided by industrial partners, Simbiose Company, Inc.) was utilized. The experiment employed a randomized block design with four replications and three treatments: (1) Control: Non-inoculation and no phosphate fertilization, (2) B0: No inoculation and (3) Co-inoculation *Bacillus* strain (B119 + B2084). Fertilization and planting procedures remained consistent with those used in the experimental single inoculation.

#### 2.4.4 Grain sampling and chemical analysis

After the maize growing period, maize ears were harvested from a stand of approximately 45 plants per plot and threshed to form a sample of the plot. This sample was then adjusted to 13% moisture content and weighed to determine the total grain weight per plot. The grains were dried at 65°C until reaching a constant mass, then milled, and the nutrient content, including phosphorus (P) content, was determined. The analysis was conducted according to the Embrapa manual (Silva, 2009). Grain yield (kg ha^−1^) was calculated based on the weight per plot, considering the area of the plot. The average yield was obtained by calculating the mean of the four replicates for each treatment. The relative yield for each treatment was calculated as the mean yield of the treatment divided by the mean yield of the B0 treatment. The results were converted to a percentage (%) by multiplying the decimal by 100, ranging from 0 to 100%. Values above 100% indicated yields higher than the controls.

#### 2.4.5 Soil sampling, chemical and enzyme analysis

Soil samples were collected from the 0–10 cm layer, with five rhizosphere soil subsamples gathered from each plot. These subsamples were then combined, sieved (2 mm), and thoroughly mixed to create a composite sample. The soil enzymatic activities of acid and alkaline phosphatase (Tabatabai, 1994), β-glucosidase (Tabatabai, 1994), and arylsulfatase (Tabatabai and Bremner, 1969) were determined in the rhizosphere soil obtained from the experiments involving co-inoculation with mixed *Bacillus* strains. Additionally, available P was assessed in Mehlich I (0.05 M HCl + 0.05 M H_2_SO_4_) extracts using the ascorbic acid blue method (Murphy and Riley, 1962).

### 2.5 Statistical analysis

The data were initially assessed for homogeneity of variances and normal distribution. Following this, a one-way ANOVA was performed, and means were compared using Duncan’s test (*p* ≤ 0.05) in R v.4.2.2 software (R Development Core Team, 2022). Furthermore, Multivariate Principal Component Analysis (PCA) tests were conducted to explore the relationships among grain yield, grain P content, available P, soil enzyme activities, and the co-inoculation treatment. These analyses were executed using Past 3.0 software (Hammer et al., 2001).

## 3 Results

### 3.1 Multifunctional mechanisms for plant growth-promoting

The strain *B. subtilis* B2084 produced siderophores, acid and alkaline phosphatases, and mineralized phytate (Table 2). Additionally, it demonstrated low production of EPS and biofilm, limited biological N fixation and moderate growth capacity in a medium with low water activity (Table 2). In both *Bacillus* strains, the total organic acids for calcium phosphate solubilization were higher than those for iron phosphate solubilization. Additionally, strain B119 exhibited a greater quantification of total organic acids compared to strain B2084. For comparative purposes, Table 2 displays references to previously published results about the characteristics of the B119 and B2084 strains.

**Table 2:** *In vitro* plant growth-promoting characteristics of *Bacillus megaterium* B119 and *B. subtilis* B2084 strains.

Characteristics*	B119	References	B2084	References
Biofilm production^1^	*++*	Velloso et al. (2020)	+	This work
Exopolysaccharide (EPS) production	*+*	Velloso et al. (2020)	+	This work
Osmotic stress tolerance	*+*	Velloso et al. (2020)	++	This work
Biological nitrogen fixation	*+*	Velloso et al. (2020)	+	This work
Acid phosphatase (μg pNP mL^−1^ h^−1^)	45.54	Velloso et al. (2020)	44.30	This work
Alkaline phosphatase (μg pNP mL^−1^ h^−1^)	57.99	Velloso et al. (2020)	68.04	This work
Total organic acid (mmol L^−1^) Fe-P solubilization	8.55	This work	3.20	This work
Total organic acid (mmol L^−1^) Ca-P solubilization	57.16	This work	40.64	This work
Siderophore type	Carboxylate	Ribeiro et al. (2018)	Carboxylate	This work
Siderophore (μM) 72 h	8.13	Ribeiro et al. (2018)	0.59	Sousa et al. (2021)
Siderophore (μM) 120 h	20.3	This work	93.5	This work
Extracellular phytase (mU mL^−1^)	3.0	Velloso et al. (2020)	2.0	This work
Intracellular phytase (mU mL^−1^)	42.0	Velloso et al. (2020)	25.00	This work
Phytate mineralization (mg L^−1^)	15.08	This work	24.66	This work
Indole acetic acid (IAA) (μg mL^−1^)	61.67	Sousa et al. (2021)	30.16	Sousa et al. (2021)
Fe-P solubilization (mg L^−1^)	39.52	Batista et al. (2018)	83.58	Batista et al. (2018)
Ca-P solubilization (mg L^−1^)	925.22	Velloso et al. (2020); Abreu et al. (2017)	120.42	Velloso et al. (2020); Abreu et al. (2017)

^*^The positive (+) and negative (−) signs mean positive and negative results for each plant growth promotion characteristic.

1 ++ Moderately, + Weakly.

**Figure 1:**
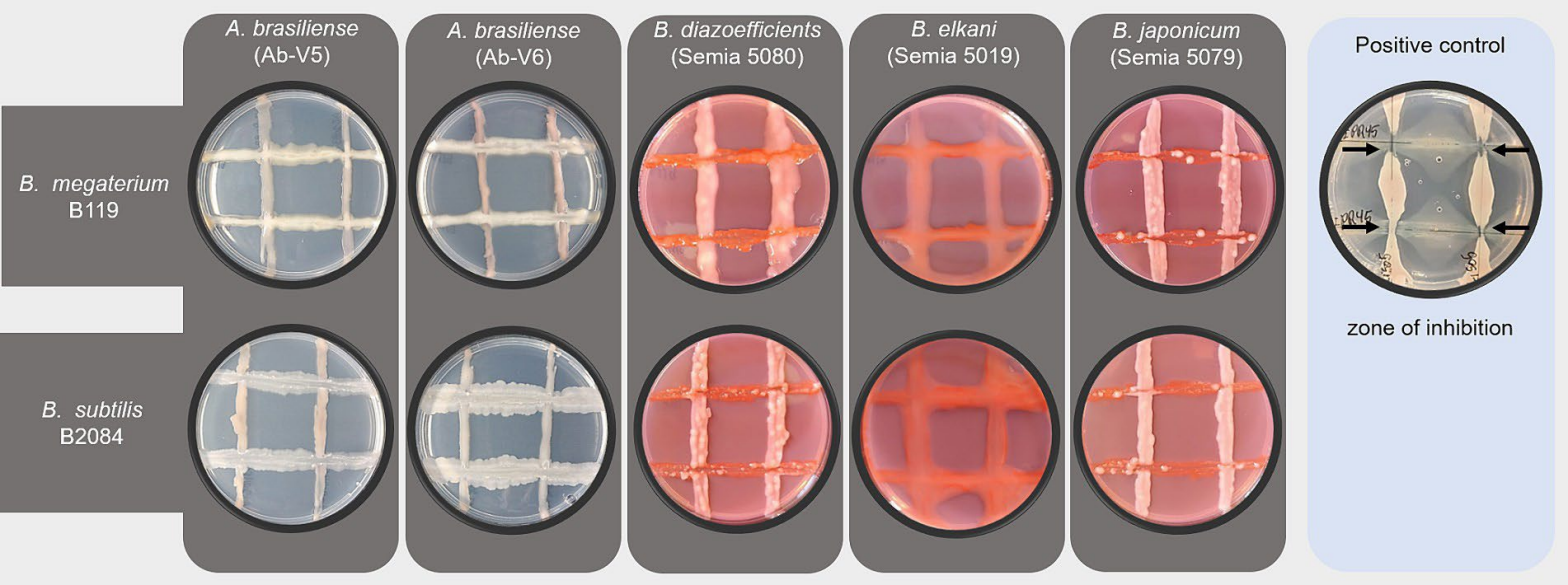
Compatibility assay among strains of *Bacillus, Bradyrizhobium and Azospirillum*. Horizontal lines on the plates represent *Bacillus* colonies and vertical lines represent colonies of *Azospirillum* or *Bradyrizhobium* strains. The positive control had strains of *Pseudomonas aeruginosa* IPR45 and *Bacillus velezensis* LIS05, with the inhibition zone indicated by black arrows.

Both strains B119 and B2084 showed compatibility with the main bacterial strains commonly used in agriculture in Brazil. The inhibition zones were not observed when *Bacillus* strains were co-cultured with *Azospirillum* or *Bradyrhizobium* strains (Figure 1).

### 3.2 Root adherence by *Bacillus* strains

**Figure 2:**
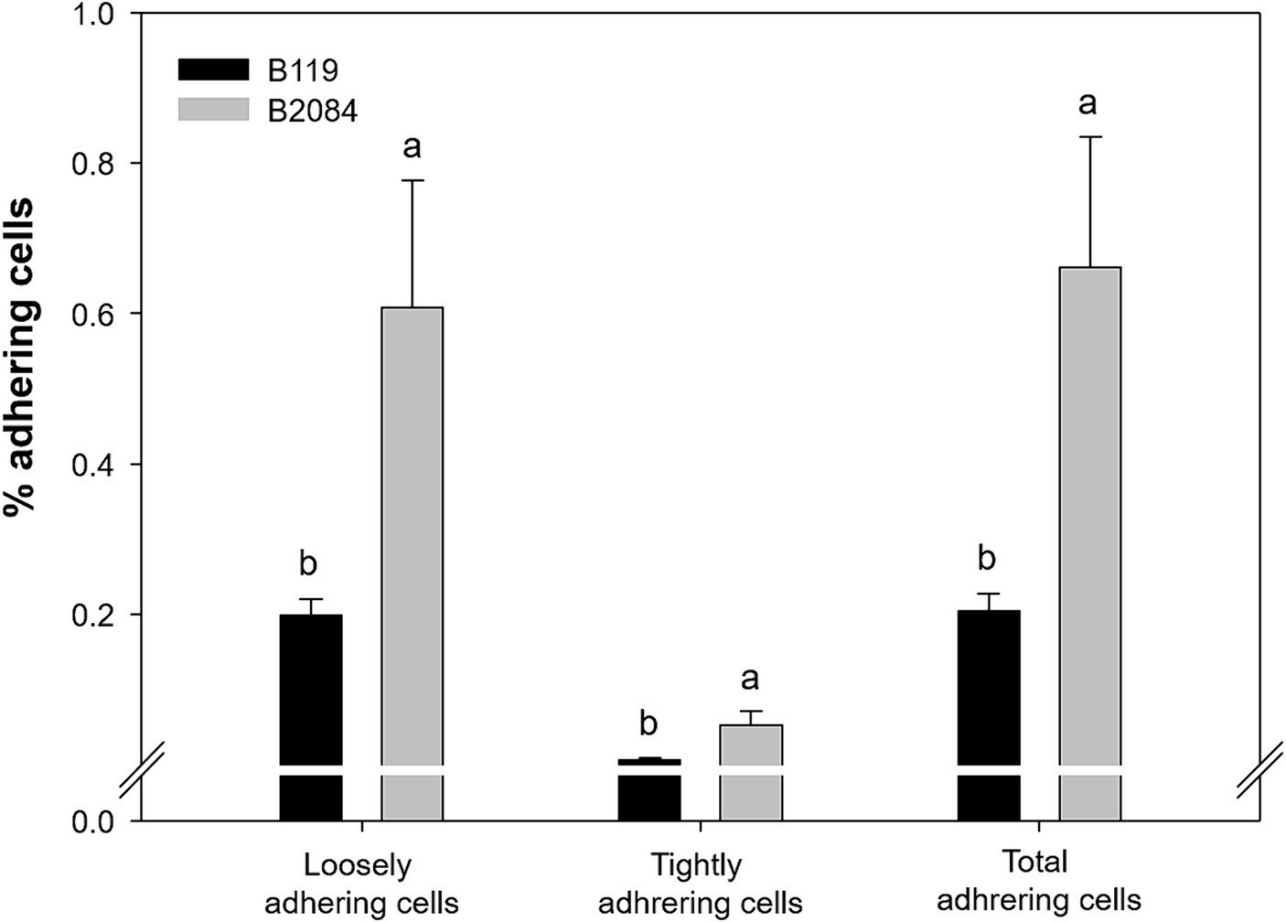
Maize root adherence by strains B119 (*Bacillus megaterium*) and B2084 (*B. subtilis*). The values represent a percentage of cells adhered to the seedling root. Means followed by different letters are significantly different (Duncan *p* ≤ 0.05).

The difference in adherence capacity between *Bacillus* strains and cells of maize roots was highly significant, with B2084 exhibiting greater adhesion than B119 (Figure 2). B2084 root adherence was 0.052% (loosely) and 0.6% (tightly adherent). In contrast, B119 showed lower cell adherence on roots, with 0.01 and 0.2% classified as tightly and loosely adherent, respectively (Figure 2).

**Figure 3:**
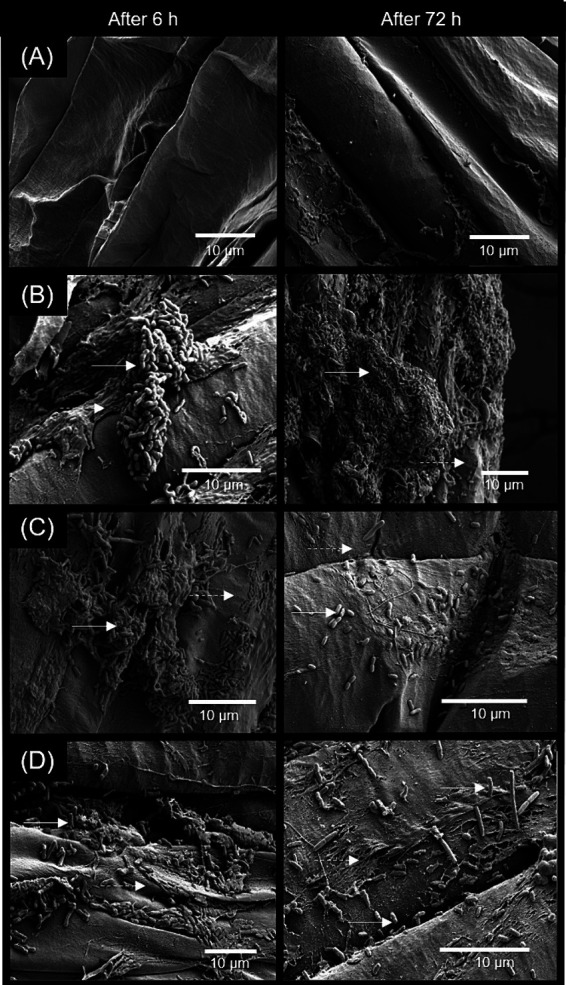
Electron microscopy analysis of maize root seedlings inoculated with *Bacillus* strains. **(A)** Control (non-inoculated); **(B)** *Bacillus megaterium* B119; **(C)** *B. subtilis* B2084; **(D)** B119 + B2084. All treatments were evaluated by 6 h and 72 h after inoculation. Full arrows indicate bacteria and dashed arrow indicate biofilm structure.

In Figure 3, electron microscopy images illustrate how *Bacillus* strains colonize maize roots through biofilm formation. The biofilm from strain B2084 covered most of the tested root, while the biofilm from strain B119 appeared more irregular.

### 3.3 Maize single inoculation with *Bacillus* strains

In Santo Antônio de Goiás, inoculation with B119 only led to increased productivity in the first season, while B2084 showed productivity increases in both the first and second season compared to the non-inoculated control. In this area, the average yield increase was approximately 6 and 3% with B119 and B2084 strain inoculations, respectively.

In Sete Lagoas, maize inoculation with both B119 and B2084 resulted in increased productivity across all three seasons compared to the non-inoculated control with no P fertilizer. Inoculation with B119 improved maize yield in the first and second seasons compared to the non-inoculated control (Table 3). Similarly, in the same area, B2084 strain inoculation increased maize productivity in the first and third seasons, differing from the non-inoculated control. On average, B119 and B2084 strain inoculations increased maize yield by 22 and 16%, respectively, compared to the non-inoculated control (Table 3).

**Table 3:** Effects of single inoculation by B119 or B2084 strains on grain P content and maize yield during three seasons (1st, 2nd and 3rd) at Santo Antônio de Goiás and Sete Lagoas, Brazil.

	Santo Antônio de Goiás					Sete Lagoas				
Treatments	1st	2nd	3rd	Mean	APR (%)***	1st	2nd	3rd	Mean	APR (%)
	Yield (kg ha^−1^)									
Control*	7,615 c	9,413 b	6,648 b	7,892 c	–	2,744 c**	1837 c	3,463 c	2,681 c	–
B0	8,013 c	9,564 b	9,251 a	8,943 b	100	7,178 b	6,751 b	6,910 b	6,886 b	100
B119	10,084 a	9,624 ab	8,878 a	9,529 a	106	8,736 a	9,058 a	7,496 b	8,430 a	122
B2084	8,688 b	10,485 a	8,572 b	9,248 ab	103	8,098 a	6,656 b	9,388 a	8,047 a	116
	Grain P content (g kg^−1^)									
Control	20.2 c	24.4 b	14.1 b	19.5 b	–	5.8 b	2.7 c	7.0 c	5.2 c	–
B0	23.5 b	23.0 b	15.5 ab	20.7 b	100	14.0 a	10.4 b	12.6 b	12.0 b	100
B119	29.0 a	26.8 a	16.0 ab	24.0 a	115	15.7 a	20.1 a	15.2 a	17.3 a	144
B2084	25.1 b	28.4 a	17.1 a	23.5 ab	113	14.3 a	12.2 b	21.0 a	15.9 a	132

^*^Control: no inoculation and zero P fertilizer; B0: no inoculation; B119 (*Bacillus megaterium*); B2084 (*B. subtilis*).

^**^Means followed by different letters in column are significantly different (Duncan *p* ≤ 0.05).

^***^APR, average productivity relative to treatment B0.

Maize grain P content was also enhanced with *Bacillus* single inoculations. In Santo Antônio de Goiás, there was an increase in grain P content when strains B119 or B2084 strains were inoculated in the second season, differing from the non-inoculated and no P fertilizer control (Table 3). In the first season, B119 differed from the non-inoculated control. Higher P grain content was observed in the second and third seasons in Sete Lagoas with the B119 treatment, and only in the third harvest with the B2084 treatment, compared to the non-inoculated control (Table 3). The inoculation of B119 and B2084 resulted in an average P increase of about 44 and 32% in Sete Lagoas, and 15 and 13% in Santo Antônio de Goiás, respectively (Table 3).

### 3.4 Co-inoculation with *Bacillus* strains

**Figure 4:**
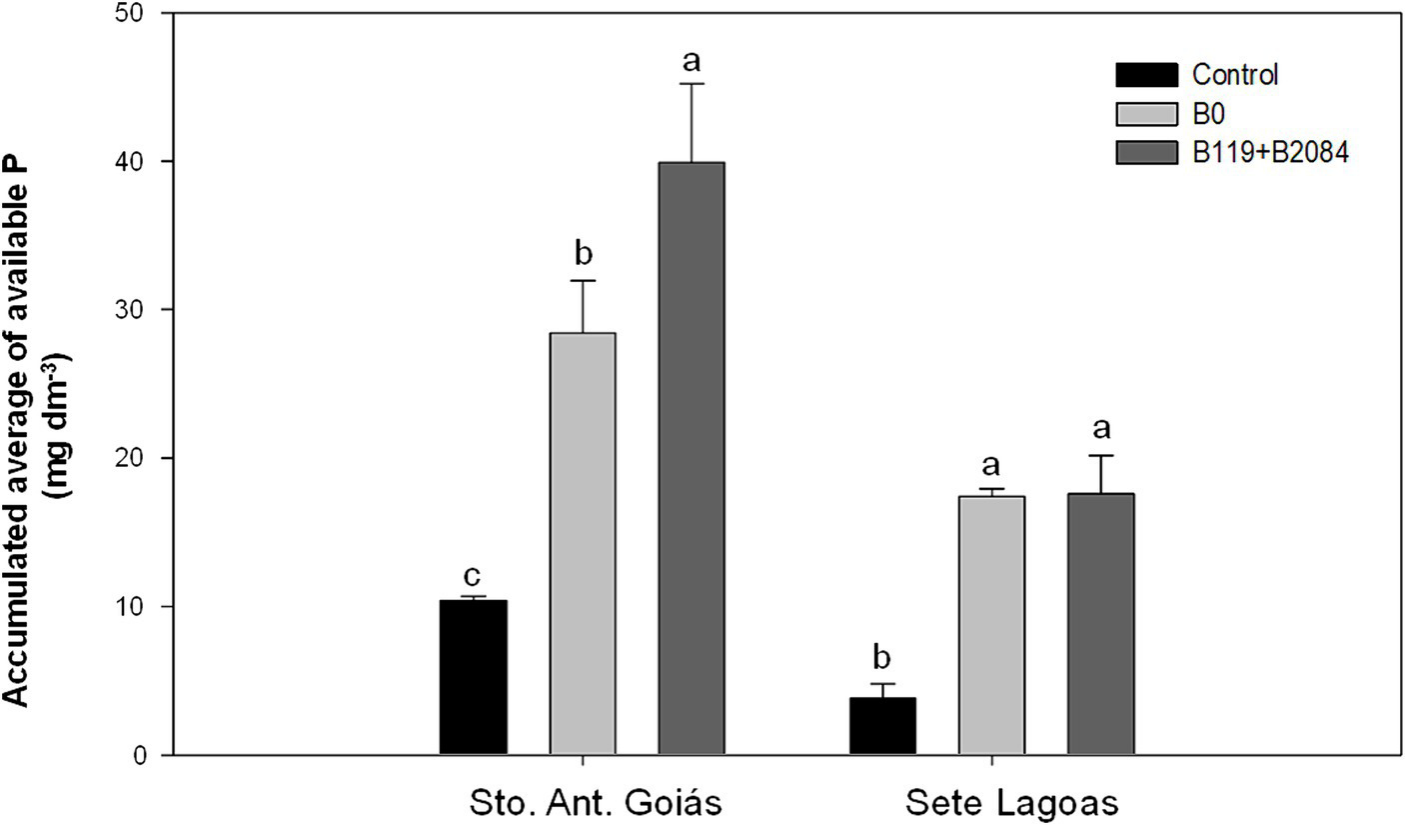
Accumulated averages of P available in the soil from areas where maize was co-inoculated with *Bacillus* strains and non-inoculated (negative control) at Santo Antônio de Goiás and Sete Lagoas, Brazil. Means followed by different letters are significantly different (Duncan *p* ≤ 0.05). Control (non-inoculation and no P fertilizer); B0 (non-inoculation); B119 + B2084 (*B. megaterium* and *B. subtilis*).

Positive results were observed across various parameters in the treatment involving both B119 and B2084 strains (Table 4; Figure 4; Supplementary Table S1). During both seasons and in both areas, maize co-inoculated with B119 + B2084 displayed higher grain yields, reaching approximately 9,621 kg ha^−1^ in Santo Antônio de Goiás and 8,593 kg ha^−1^ in Sete Lagoas (Table 4). Compared to the B0 treatment, there was an average increase in productivity of around 11 and 24% in Santo Antônio de Goiás and Sete Lagoas, respectively (Table 4).

**Table 4:** Effects of co-inoculation of B119 and B2084 strains on grain P content and maize yield during two seasons (1st and 2nd) at Santo Antônio de Goiás and Sete Lagoas, Brazil.

Treatments	Santo Antônio de Goiás				Sete Lagoas			
	1st	2nd	Mean	APR (%)***	1st	2nd	Mean	APR (%)
	Yield (kg ha^−1^)							
Control*	8,302 c**	7,030 c	7,666 c	–	3,228 c	2,333 c	2,781 c	–
B0	9,413 b	7,970 b	8,692 b	100	8,071 b	5,832 b	6,952 b	100
B119 + B2084	10,587 a	8,656 a	9,622 a	111	10,415 a	6,770 a	8,593 a	124
	Grain P content (g kg^−1^)							
Control	31.5 b	22.7 b	34.1 b	–	10.5 c	8.1 b	9.3 c	–
B0	35.7 b	25.7 b	38.7 b	100	26.2 b	20.1 a	23.2 b	100
B119 + B2084	45.5 a	38.3 a	43.5 a	112	33.1 a	22.6 a	27.9 a	120

^*^Control: no inoculation and zero P fertilizer; B0: no inoculation; B119 (*Bacillus megaterium*); B2084 (*B. subtilis*).

^**^Means followed by different letters in column are significantly different (Duncan *p* ≤ 0.05).

^***^APR, average productivity relative to treatment B0.

Co-inoculation led to increased grain P content in two seasons in Santo Antônio de Goiás, but only in the first harvest in Sete Lagoas. Additionally, co-inoculation showed higher average P accumulation in grains compared to the B0 and control treatments (Table 4), with increases of 20 and 12% over the B0 treatment in Sete Lagoas and Santo Antônio de Goiás, respectively. Moreover, a higher accumulated mean of soil available P was observed in the co-inoculation treatment in Santo Antônio de Goiás, differing from the B0 treatment (Figure 4).

**Figure 5:**
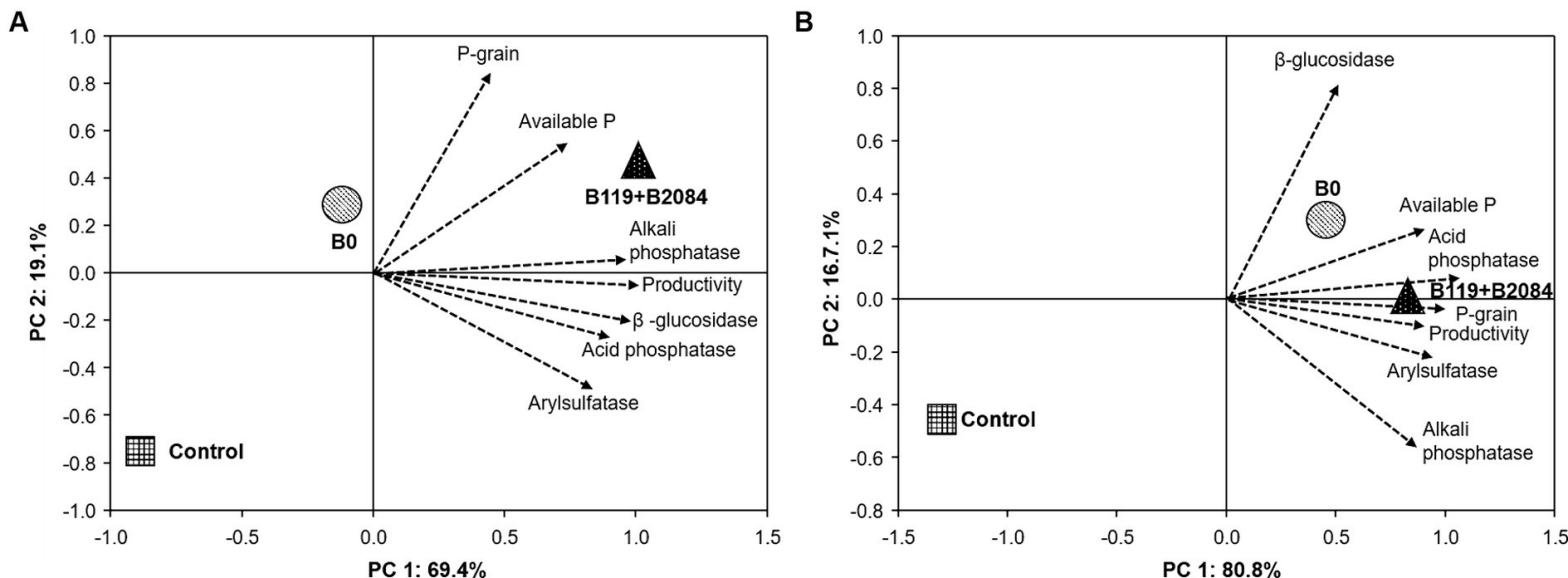
Principal Component Analysis (PCA) of the average of all traits evaluated at Santo Antonio de Goiás **(A)** and Sete Lagoas **(B)**, Brazil. Arrows represent the correlation among traits. Circle, square and triangles are centroids of the mean scores of the treatments. Control (non-inoculation and no P fertilizer), B0 (non-inoculated) and B119 + B2084 (*Bacillus megaterium* and *B. subtilis*), respectively. Productivity = accumulated average yield; P-grain = accumulated average P in the maize grain; Available P = accumulated average of P available in the soil.

The enzymatic activities of phosphatases and β-glucosidase increased in treatments involving B119 + B2084. Acid phosphatase was stimulated in the first season in Sete Lagoas and in the second season in Santo Antônio de Goiás (Supplementary Table S1). Only maize co-inoculated in Santo Antônio de Goiás exhibited higher alkaline phosphatase and β-glucosidase activities in the soil during both seasons. The activity of arylsulfatase was not affected by the treatments. The PCA effectively distinguished treatments in the factorial space (Figure 5) based on the average of both periods in the co-inoculation field test. In Santo Antônio de Goiás, PC1 explained 69.4% of the variation and clearly separated B119 + B2084 from the B0 and control treatments (Figure 5A). In Sete Lagoas, there was closer proximity among treatments B119 + B2084 and B0 (Figure 5B). In this area, PC1 explained 80.8% of the variation and separated B119 + B2084 and B0 from the control treatment. It was observed that the mixed *Bacillus* strains exhibited strong correlations with several response variables in both areas, such as productivity, P-grain content, acid and alkaline phosphatase, available P, β-glucosidase, and arylsulfatase.

## 4 Discussion

The mechanisms underlying plant growth promotion by *B. megaterium* B119 and *B. subtilis* B2084 were characterized, with results complementing previously published research by our group (Ribeiro et al., 2018; Velloso et al., 2020; Sousa et al., 2021). We highlighted the plant growth-promoting properties of these two *Bacillus* strains, particularly B2084, through extensive field trials conducted in two regions characterized by distinct edaphic and climatic conditions, as well as varying soil P levels, typical of the Brazilian Cerrado.

Both strains, B119 and B2084, exhibited multifunctional mechanisms suitable for use as inoculants, including phosphate solubilization and mineralization, as well as the production of IAA-like compounds, siderophores, EPS, and biofilms (Table 2). These mechanisms, particularly those related to P mobility, play a crucial role in enhancing nutrient uptake and promoting plant development, especially in soils with high P adsorption capacity (Richardson and Simpson, 2011; Mahmood et al., 2016; Kalayu, 2019; Tian et al., 2021). EPS and biofilm production have been associated with numerous benefits for plants, including biocontrol, heavy metal accumulation in the soil, enhanced tolerance to water scarcity and salinity, and bacterial adhesion to roots (Radhakrishnan et al., 2017; Saxena et al., 2020; Knights et al., 2021). It is likely that the ability of strains B119 and B2084 to adhere to maize roots was facilitated by their production of EPS and biofilms, which serve as the primary mode of adhesion for gram-positive bacteria to roots (Beauregard et al., 2013; Knights et al., 2021). However, differences in polymer composition between bacterial species may influence their adhesion to root surfaces due to variations in electrostatic and hydrophobic properties (Beauregard et al., 2013; Habib et al., 2017; Knights et al., 2021). Therefore, the disparity in root adhesion between B119 and B2084 could be attributed to differences in the composition of their EPS and biofilms. Once root colonization is established, *Bacillus* strains are more likely to perform their beneficial functions for plant growth. Among these functions, phosphate solubilization and mineralization play key roles in improving the P cycle by rendering P soluble in the soil, primarily through the action of organic acids and other mechanisms. Previous studies by Abreu et al. (2017) and Batista et al. (2018) investigated the solubilization of Ca and Fe phosphates by strains B119 and B2084, respectively. Their findings revealed that B2084 was highly effective in solubilizing calcium phosphate but had a limited capacity for dissolving iron phosphate compared to strain B119 (Abreu et al., 2017; Batista et al., 2018; Velloso et al., 2020).

Unlike Ca phosphate, Fe phosphate is more challenging to dissolve due to the requirement of a pH lower than 2.0–2.5 for solubilization, whereas Ca phosphate solubilization occurs at a pH of 2.5–4.0 (Tian et al., 2021). Hence, other mechanisms such as siderophores and EPS may contribute to the solubilization of Fe phosphate through metal chelation (Mahmood et al., 2016; Radhakrishnan et al., 2017; Costa et al., 2018). Both *Bacillus* strains were capable of producing carboxylate-type siderophores, with B2084 demonstrating a higher capacity, showcasing the physiological adaptability of these strains to different phosphate forms. Carboxylate-type siderophores, produced by a limited number of bacteria like *Rhizobium meliloti,* possess carboxyl and hydroxyl groups for iron acquisition (Khan et al., 2018), thereby aiding in the release of P bound to Fe (Ghosh et al., 2015; Tian et al., 2021; Cui et al., 2022). For instance, Cui et al. (2022) observed that *Streptomyces* sp. (CoT10) chelates and releases P bound to Fe with the assistance of siderophores. Therefore, the production of siderophores and EPS are crucial mechanisms involved in phosphate solubilization by strains B119 and B2084, although other mechanisms such as inorganic acids and proton efflux may also play a role (Mahmood et al., 2016; Costa et al., 2018; Kalayu, 2019). Furthermore, the strain B2084 demonstrated the ability to release P from phytate through phosphatase and phytase activities, which may also be present in B119 (Velloso et al., 2020). Phytate, representing about 60% of total organic P, is a major source of P in soil (Singh and Satyanarayana, 2011). Phytase cleaves phytate into smaller phosphomonoester fragments, subsequently degraded by phosphatases, thereby releasing P (Pang and Kolenko, 1986; Richardson and Simpson, 2011). Thus, strains B119 and B2084 have the potential to access organic P sources, which constitute a significant P pool in most soils but are typically resistant to degradation.

It is important to note that both *Bacillus* strains may access P contained in various fertilizers used in agriculture, including Ca (calcium phosphates and calcareous), as well as natural P present in iron and aluminum oxides and organic matter in tropical soils. Many mechanisms of phosphate solubilization and mineralization exhibited by strains B119 and B2084 may mitigate P adsorption in tropical soils, where soluble P reacts with clay minerals and metal ions (Ca, Fe, Al) to form poorly soluble complexes that are unavailable to plants (Hinsinger, 2001; Kalayu, 2019; Pavinato et al., 2020). This represents a significant strategy for utilizing these strains as inoculants. Under field conditions, there was a consistent increase in maize growth parameters across all crop seasons with either single or co-inoculation. Overall, the co-inoculation with *Bacillus* strains outperformed productivity values and exhibited higher P accumulation in the grain compared to the non-inoculated treatment (Table 4). The relative yield also saw an increase in both areas. Numerous studies have highlighted the effectiveness of co-inoculation in promoting plant growth (Emami et al., 2020; Pereira et al., 2020; Guimarães et al., 2021; Leite et al., 2022; Ribeiro et al., 2022; Santos et al., 2022). For instance, Emami et al. (2020) observed enhanced P absorption and growth of wheat cultivars when inoculated with a mixture of rhizospheric and endophytic phosphate-solubilizing microbes in P-deficient soil. Similarly, Leite et al. (2022) reported increased productivity and profitability in soybeans co-inoculated with B119 and B2084 alongside arbuscular mycorrhizal fungus. Co-inoculation with *Bacillus* species and *Azospirillum* sp. also demonstrated positive effects on maize plants by promoting root development, increasing available P in the soil, and stimulating phosphatase activities (Ribeiro et al., 2022). In addition, enzyme activities serve as reliable indicators of soil quality and organic matter mineralization (Cardoso et al., 2013). The positive relationship observed between mixed *Bacillus* strains inoculation and the activity of β-glucosidase and phosphatase suggests an enhancement in organic matter mineralization (Cardoso et al., 2013; Elhaissoufi et al., 2022). For example, alkaline phosphatase is an enzyme exclusively produced by soil microorganisms and may aid in releasing P from soil organic matter. Additionally, β-glucosidase is associated with the breakdown of cellulose, the primary structural component in plants (Elhaissoufi et al., 2022). In Santo Antônio de Goiás, both enzymes exhibited high activity in the soil treated with B119 + B2084, which was different from the control treatments. This demonstrates the positive impact of *Bacillus* inoculation on soil quality and carbon and nutrient cycling.

Single inoculation with B119 or B2084 strains did not consistently increase maize productivity in each harvest, particularly B2084. In Santo Antônio do Goiás, Sousa et al. (2021) noted no difference in maize productivity between plants inoculated with B2084 strains and those without inoculation treatment. Consistent with our findings, the literature indicates that the B119 strain exhibits a greater ability to increase grain yield in soils with high P levels compared to the B2084 strains, especially when fertilized with TSP (Sousa et al., 2021; Santos et al., 2022). The positive impact observed in both studied areas indicates the potential use of co-inoculation with two *Bacillus* strains in soils with varying conditions and initial P levels, ranging from medium to high (Goiás) to low (Sete Lagoas). In low-P soil fertility such as Sete Lagoas, co-inoculation led to a significant increase in grain productivity and accumulated P, even though the available P did not differ from the conventional treatment with TSP fertilization (Figure 2). In such soils, where rapid P adsorption and plant absorption occur, co-inoculation with *Bacillus* enhanced P solubilization and improved P utilization by maize, resulting in elevated grain P content. For instance, Leggett et al. (2015) suggested greater efficiency of the inoculant *Penicillium bilaiae* in maize crops grown in low-fertility soils. Conversely, in soils with built fertility such as Santo Antônio de Goiás, there was an 11% increase in relative yield with co-inoculation (Tables 3, 4). These soils, with a history of successive fertilizer applications, exhibit cumulative residual effects that enhance certain chemical fertility attributes. In such cases, mixed *Bacillus* inoculation may maximize productivity and P content in the grain, in addition to increasing available P in the soil of Santo Antônio de Goiás.

The combination of phosphate solubilization, mineralization mechanisms, and IAA-like production likely could help explain the results observed in the field trials. Both *Bacillus* strains produce high levels of the phytohormone IAA-like, particularly B119, known to stimulate root growth (Sousa et al., 2021). Enhanced root growth may increase root surface area, facilitating the acquisition of available P in the soil. Numerous plant species have exhibited improved root growth and development after inoculation with IAA-producing bacteria (Mohite, 2013; Bahadir et al., 2018; Sousa et al., 2021). The modified root structure of maize likely led to better soil exploration, resulting in areas with higher P availability and other nutrients, thereby increasing P accumulation in the grain and improving productivity. This underscores the significant positive effects of P solubilizing bacteria inoculation on maize productivity and nutrient uptake, with implications for reducing phosphate fertilizer usage in the field (Rajapaksha et al., 2011; Granada et al., 2018; Patil et al., 2021).

The ecological aspects of bacterial co-inoculation in plants complement their physiological characteristics and ecological niches. Both *Bacillus* strains, with distinct origins (*B. megaterium* B119 from rhizosphere and *B. subtilis* B2084 from endophytic bacteria), exhibit different and similar properties *in vitro* tests. The genus *Bacillus*, easily isolated from maize, suggests an evolutionary relationship and greater adaptability when used as a maize inoculant. The competitive ability and adaptability of these strains to soil endogenous microbes likely contribute to their efficacy as plant growth promoters in field trials. Moreover, *Bacillus* species’ high adaptability and forming endospores resistant to temperature and drought confers advantages in commercial production due to extended shelf life, unlike other phosphate-solubilizing microbes such as *Pseudomonas*.

## 5 Conclusion

The results demonstrate that strains *B. megaterium* B119 and *B. subtilis* B2084 represent valuable technological resources for maize cultivation, promoting sustainable agriculture and reducing costs associated with chemical fertilization. The development of the first Brazilian-made inoculant for P solubilization highlights the efficiency of co-inoculation with *Bacillus* strains in promoting plant growth and maximizing P utilization through various biological mechanisms.

## Data availability statement

The raw data supporting the conclusions of this article will be made available by the authors, without undue reservation.

## Author contributions

CO-P: Conceptualization, Data curation, Funding acquisition, Project administration, Supervision, Visualization, Writing – original draft, Writing – review & editing, Investigation, Methodology. DB: Data curation, Visualization, Writing – original draft, Writing – review & editing, Conceptualization, Formal analysis, Methodology. SS: Data curation, Visualization, Writing – original draft, Writing – review & editing, Methodology, Conceptualization. VR: Data curation, Methodology, Writing – original draft, Investigation, Conceptualization. FCS: Methodology, Visualization, Writing – original draft, Investigation. UP: Formal analysis, Methodology, Writing – review & editing. FFS: Methodology, Writing – original draft. EG: Visualization, Writing – original draft, Writing – review & editing, Methodology, Conceptualization. IM: Data curation, Methodology, Visualization, Writing – original draft, Conceptualization.

## References

[ref1] Abreu C. S. Figueiredo J. E. F. Oliveira C. A. Santos V. L. Gomes E. A. Ribeiro V. P.. (2017). Maize endophytic bacteria as mineral phosphate solubilizers. Genet. Mol. Res. 16:gmr16019294. doi: 10.4238/gmr16019294, PMID: 28218783

[ref2] Agrolink. (2023). Mais de 70% dos fertilizantes são importados no Brasil. Available at: https://www.agrolink.com.br/noticias/mais-de-70--dos-fertilizantes-sao-importados-no-brasil_483200.html/ (Accessed March 15, 2024).

[ref3] Albuquerque P. E. P. (2007). Planilha eletrônica para programação da irrigação em sistemas de aspersão convencional, pivô central e sulcos. Available at: http://www.infoteca.cnptia.embrapa.br/infoteca/handle/doc/482079 (Accessed March 15, 2024).

[ref4] Aloo B. N. Makumba B. A. Mbega E. R. (2018). The potential of Bacilli rhizobacteria for sustainable crop production and environmental sustainability. Microbiol. Res. 219, 26–39. doi: 10.1016/j.micres.2018.10.011 30642464

[ref5] Alori E. T. Glick B. R. Babalola O. O. (2017). Microbial phosphorus solubilization and its potential for use in sustainable agriculture. Front. Microbiol. 8:971. doi: 10.3389/fmicb.2017.00971 28626450 PMC5454063

[ref6] Arora N. K. Verma M. (2017). Modified microplate method for rapid and efficient estimation of siderophore produced by bacteria. 3 Biotech 7:381. doi: 10.1007/s13205-017-1008-y PMC5658296 29109926

[ref7] Bahadir P. S. Liaqat F. Eltem R. (2018). Plant growth promoting properties of phosphate solubilizing Bacillus species isolated from the Aegean region of Turkey. Turk. J. Bot. 42, 183–196. doi: 10.3906/bot-1706-51

[ref8] Batista F. C. Fernandes T. A. Abreu C. S. Oliveira M. C. Ribeiro V. P. Gomes E. A.. (2018). Potencial de microrganismos rizosféricos e endofíticos de milho em solubilizar o fosfato de ferro e produzir sideróforos. Available at: https://ainfo.cnptia.embrapa.br/digital/bitstream/item/183975/1/bol-166.pdf (Accessed March 15, 2024).

[ref9] Beauregard P. B. Chai Y. Vlamakis H. Losick R. Kolter R. (2013). *Bacillus subtilis* biofilm induction by plant polysaccharides. Proc. Natl. Acad. Sci. USA 110, E1621–E1630. doi: 10.1073/pnas.1218984110, PMID: 23569226 PMC3637697

[ref10] Biedendieck R. Knuuti T. Moore S. J. Jahn D. (2021). The “beauty in the beast”—the multiple uses of Priestia megaterium in biotechnology. Appl. Microbiol. Biotechnol. 105, 5719–5737. doi: 10.1007/s00253-021-11424-6, PMID: 34263356 PMC8390425

[ref11] Cardoso E. J. B. N. Vasconcellos R. L. F. Bini D. Miyauchi M. Y. H. Santos C. A. Alves P. R. L.. (2013). Soil health: looking for suitable indicators. What should be considered to assess the effects of use and management on soil health? Sci. Agric. 70, 274–289. doi: 10.1590/S0103-90162013000400009

[ref12] Costa O. Y. A. Raaijmakers J. M. Kuramae E. E. (2018). Microbial extracellular polymeric substances: ecological function and impact on soil aggregation. Front. Microbiol. 23:1636. doi: 10.3389/fmicb.2018.01636 PMC6064872 30083145

[ref13] Cui K. Xu T. Chen J. Yang H. Liu X. Zhuo R.. (2022). Siderophores, a potential phosphate solubilizer from the endophyte Streptomyces sp. CoT10, improved phosphorus mobilization for host plant growth and rhizosphere modulation. J. Clean. Prod. 367:133110. doi: 10.1016/j.jclepro.2022.133110

[ref14] Döbereiner J. (1989). “Isolation and identification of root associated diazotrophs” in Nitrogen fixation with non-legumes. Developments in plant and soil sciences. eds. Skinner F. A. Boddey R. M. Fendrik I. (Dordrecht: Springer), 103–108.

[ref15] Dworkin M. Falkow S. Rosenberg E. Schleifer K.-H. Stackebrandt E. (2006). The prokaryotes. A handbook on the biology of bacteria. New York: Springer-Verlag.

[ref16] Elhaissoufi W. Ghoulam C. Barakat A. Zeroual Y. Bargaz A. (2022). Phosphate bacterial solubilization: a key rhizosphere driving force enabling higher P use efficiency and crop productivity. J. Adv. Res. 38, 13–28. doi: 10.1016/j.jare.2021.08.014, PMID: 35572398 PMC9091742

[ref17] Emami S. Alikhani H. A. Pourbabaee A. A. Etesami H. Motasharezadeh B. Sarmadian F. (2020). Consortium of endophyte and rhizosphere phosphate solubilizing bacteria improves phosphorous use efficiency in wheat cultivars in phosphorus deficient soils, rhizosphere. Rhizosphere 14, 2452–2198. doi: 10.1016/j.rhisph.2020.1001961

[ref18] Ghosh P. Rathinasabapathi B. Ma L. Q. (2015). Phosphorus solubilization and plant growth enhancement by arsenic-resistant bacteria. Chemosphere 134, 1–6. doi: 10.1016/j.chemosphere.2015.03.048, PMID: 25880602

[ref19] Gomes E. A. Silva C. U. Marriel I. E. Oliveira C. A. Lana U. P. (2014). Rock phosphate solubilizing microorganisms isolated from maize rhizosphere soil. Rev. Bras. Milho Sorgo. 13, 69–81. doi: 10.18512/1980-6477/rbms.v13n1p69-81

[ref20] Granada C. E. Passaglia L. M. P. Souza E. M. Sperotto R. A. (2018). Is phosphate solubilization the forgotten child of plant growth-promoting rhizobacteria? Front. Microbiol. 9:2054. doi: 10.3389/fmicb.2018.02054 30233533 PMC6129612

[ref21] Greiner R. (2007). “Phytate-degrading enzymes: regulation of synthesis in microorganisms and plants” in Inositol phosphates: Linking agriculture and the environment/secondary Phytate-degrading enzymes: Regulation of synthesis in microorganisms and plants. eds. Turner B. L. Richardson A. E. Mullaney E. J. (Oxfordshire: CAB International), 78–96.

[ref22] Guimarães V. F. Klein J. Silva A. S. L. Klein D. K. (2021). Eficiência de inoculante contendo *Bacillus megaterium* (B119) e Bacillus subitilis (B2084) para a cultura do milho, associado à fertilização fosfatada. Rev. Caribeña Cienc. Soc. 12, 3250–3287. doi: 10.55905/rcssv12n7-016

[ref23] Gupta R. S. Patel S. Saini N. Chen S. (2020). Robust demarcation of 17 distinct Bacillus species clades, proposed as novel Bacillaceae genera, by phylogenomics and comparative genomic analyses: description of Robertmurraya kyonggiensis sp. nov. and proposal for an emended genus Bacillus limiting it only to the members of the subtilis and Cereus clades of species. Int. J. Syst. Evol. Microbiol. 70, 5753–5798. doi: 10.1099/ijsem.0.004475, PMID: 33112222

[ref24] Habib C. Yu Y. Gozzi K. Ching C. Shemesh M. Chai Y. (2017). Characterization of the regulation of a plant polysaccharide utilization operon and its role in biofilm formation in *Bacillus subtilis*. PLoS One 12:e0179761. doi: 10.1371/journal.pone.0179761, PMID: 28617843 PMC5472308

[ref25] Hammer Ø. Harper D. A. T. Ryan P. D. (2001). PAST: Paleontological statistics software package for education and data analysis. Palaeontologia Electronica.

[ref26] Hinsinger P. (2001). Bioavailability of soil inorganic P in the rhizosphere as affected by root-induced chemical changes: a review. Plant Soil 273, 173–195. doi: 10.1023/A:1013351617532

[ref27] Hozore E. Alexander M. (1991). Bacterial characteristic important to rhizosphere competence. Soil Biol. Biochem. 23, 717–723. doi: 10.1016/0038-0717(91)90140-F, PMID: 38743972

[ref28] Javed S. Javaid A. Hanif U. Bahadur S. Sultana S. Shuaib M.. (2021). Effect of necrotrophic fungus and PGPR on the comparative histochemistry of *Vigna radiata* by using multiple microscopic techniques. Microsc. Res. Tech. 84, 2737–2748. doi: 10.1002/jemt.23836, PMID: 34028133

[ref29] Kalayu G. (2019). Phosphate solubilizing microorganisms: promising approach as biofertilizers. Int. J. Agron. 2019:4917256. doi: 10.1155/2019/4917256

[ref30] Karnovsky M. J. (1965). A formaldehyde-glutaraldehyde fixative of high osmolality for use in electron-microscopy. J. Cell Biol. 27, 137–138.

[ref31] Khan A. Singh P. Srivastava A. (2018). Synthesis, nature and utility of universal iron chelator – Siderophore: a review. Microbiol. Res. 212-213, 103–111. doi: 10.1016/j.micres.2017.10.012, PMID: 29103733

[ref32] Khan M. S. Zaidi A. Wani P. A. (2007). Role of phosphate-solubilizing microorganisms in sustainable agriculture: a review. Agron. Sustain. Dev. 27, 29–43. doi: 10.1051/agro:2006011

[ref33] Knights H. E. Jorrin B. Haskett T. L. Poole P. S. (2021). Deciphering bacterial mechanisms of root colonization. Environ. Mcrobiol. Rep. 13, 428–444. doi: 10.1111/1758-2229.12934 PMC8651005 33538402

[ref34] Leggett M. Newlands N. K. Greenshields D. West L. Inman S. Koivunen M. E. (2015). Maize yield response to a phosphorus-solubilizing microbial inoculant in field trials. J. Agric. Sci. 153, 1464–1478. doi: 10.1017/S0021859614001166, PMID: 26500375 PMC4611360

[ref35] Leite R. C. Pereira Y. C. Oliveira-Paiva C. A. Moraes A. J. G. Silva G. B. (2022). Increase in yield, leaf nutrient, and profitability of soybean co-inoculated with Bacillus strains and Arbuscular mycorrhizal fungi. Rev. Bras. Cienc. Solo. 46:e0220007. doi: 10.36783/18069657rbcs20220007

[ref36] Lertcanawanichakul M. Sawangnop S. (2008). A comparison of two methods used for measuring the antagonistic activity of Bacillus species, Walailak. J. Sci. Tech. 5, 161–171.

[ref37] Mahmood S. Daur I. Al-Solaimani S. G. Ahmad S. Madkour M. H. Yasir M.. (2016). Plant growth promoting rhizobacteria and silicon synergistically enhance salinity tolerance of mung bean. Front. Plant Sci. 7:876. doi: 10.3389/fpls.2016.00876, PMID: 27379151 PMC4911404

[ref38] Mohite B. (2013). Isolation and characterization of indole acetic acid (IAA) producing bacteria from rhizospheric soil and its effect on plant growth. J. Soil Sci. Plant Nutr. 13, 638–649. doi: 10.4067/S0718-95162013005000051

[ref39] Murphy J. Riley J. P. (1962). A modified single solution method for the determination of phosphate in natural waters. Analyt. Chim. Acta. 27, 31–36. doi: 10.1016/S0003-2670(00)88444-5, PMID: 14982454

[ref40] Nahas E. Centurion J. F. Assis L. C. (1994). Microrganismos solubilizadores de fosfatos e produtores de fosfatases de vários solos. Rev. Bras. Cienc. Solo. 18, 43–48.

[ref41] Nautiyal C. S. (1999). An efficient microbiological growth medium for screening phosphate solubilizing microorganisms. FEMS Microbiol. Lett. 170, 265–270. doi: 10.1111/j.1574-6968.1999.tb13383.x, PMID: 9919677

[ref42] O’Callaghan M. Ballard R. A. Wright D. (2022). Soil microbial inoculants for sustainable agriculture: limitations and opportunities. Soil Use Manag. 38, 1340–1369. doi: 10.1111/sum.12811, PMID: 36687657

[ref43] Oliveira C. A. Alves V. M. C. Marriel I. E. Gomes E. A. Scotti M. R. Carneiro N. P.. (2009). Phosphate solubilizing microorganisms isolated from rhizosphere of maize cultivated in an oxisol of the Brazilian Cerrado biome. Soil Biol. Biochem. 41, 1782–1787. doi: 10.1016/j.soilbio.2008.01.012

[ref44] Pang P. C. K. Kolenko H. (1986). Phosphomonoesterase activity in forest soils. Soil Biol. Biochem. 18, 35–39. doi: 10.1016/0038-0717(86)90100-8, PMID: 38678896

[ref45] Parte A. C. Sardà Carbasse J. Meier-Kolthoff J. P. Reimer L. C. Göker M. (2020). List of prokaryotic names with standing in nomenclature (LPSN) moves to the DSMZ. Int. J. Syst. Evol. Micr. 70, 5607–5612. doi: 10.1099/ijsem.0.004332, PMID: 32701423 PMC7723251

[ref9001] Patil P. M. Kuligod V. B. Hebsur N. S. Patil C. R. Kulkarni G. N. (2012). Effect of phosphate solubilizing fungi and phosphorus levels on growth, yield and nutrient content in maize (Zea mays). Karnataka J. Agric. Sci. 25, 58–62.

[ref46] Patil P. M. Kuligod V. B. Hebsur N. S. Patil C. R. Kulkarni G. N. (2021). Effect of phosphate solubilizing fungi and phosphorus levels on growth, yield and nutrient content in maize (*Zea mays*). Karnataka J. Agric. Sci. 25, 58–62.

[ref47] Paulo E. M. Vasconcelos M. P. Oliveira I. S. Affe H. M. J. Nascimento R. Melo I. S.. (2012). An alternative method for screening lactic acid bacteria for the production of exopolysaccharides with rapid confirmation. Cienc. Tecnol. Aliment. 32, 710–714. doi: 10.1590/S0101-2061201200500009

[ref48] Pavinato P. S. Cherubin M. R. Soltangheis A. Rocha G. C. Chadwick D. R. Jones D. L. (2020). Revealing soil legacy phosphorus to promote sustainable agriculture in Brazil. Sci. Rep. 10:15615. doi: 10.1038/s41598-020-72302-1 32985529 PMC7522976

[ref49] Pavinato P. S. Rosolem C. A. (2008). Disponibilidade de nutrientes no solo: decomposição e liberação de compostos orgânicos de resíduos vegetais. Ver. Bras. Cienc. Solo. 32, 911–920. doi: 10.1590/S0100-06832008000300001

[ref50] Pereira N. C. M. Galindo F. S. Gazola R. P. D. Dupas E. Rosa P. A. L. Mortinho E. S.. (2020). Corn yield and phosphorus use efficiency response to phosphorus rates associated with plant growth promoting bacteria. Front. Env. Sci. 8:512530. doi: 10.3389/fenvs.2020.00040

[ref51] R Development Core Team. (2022). R: a language and environment for statistical computing. R foundation for statistical computing. Available at: https://www.r-project.org/ (Accessed March 15, 2024).

[ref52] Radhakrishnan R. Hashem A. Abd Allah E. F. (2017). Bacillus: a biological tool for crop improvement through bio-molecular changes in adverse environments. Front. Physiol. 8:667. doi: 10.3389/fphys.2017.00667 28932199 PMC5592640

[ref53] Rajapaksha R. M. C. P. Herath D. Senanayake A. P. Senevirathne M. G. T. L. (2011). Mobilization of rock phosphate phosphorus through bacterial inoculants to enhance growth and yield of wetland rice. Commun. Soil Sci. Plant Anal. 42, 301–314. doi: 10.1080/00103624.2011.539084

[ref54] Ribeiro V. P. Gomes E. A. Sousa S. M. Lana U. G. P. Coelho A. M. Marriel I. E.. (2022). Co-inoculation with tropical strains of Azospirillum and Bacillus is more efficient than single inoculation for improving plant growth and nutrient uptake in maize. Arch. Microbiol. 204:143. doi: 10.1007/s00203-022-02759-3 35044594

[ref55] Ribeiro V. P. Marriel I. E. Sousa S. M. Lana U. G. P. Mattos B. B. Oliveira-Paiva C. A.. (2018). Endophytic Bacillus strains enhance pearl millet growth and nutrient uptake under low-P. Braz. J. Microbiol. 49S, 40–46. doi: 10.1016/j.bjm.2018.06.005 PMC6328806 30150087

[ref56] Richardson A. E. Simpson R. J. (2011). Soil microorganisms mediating phosphorus availability update on microbial phosphorus. Plant Physiol. 156, 989–996. doi: 10.1104/pp.111.175448, PMID: 21606316 PMC3135950

[ref57] Santos F. C. dos Reis D. P. Gomes E. A. Ladeira D. A. de Oliveira A. C. Melo I. G.. (2022). Influence of phosphorus-solubilizing microorganisms and phosphate amendments on pearl millet growth and nutrient use efficiency in different soils types. Afr. J. Microbiol. Res. 16, 95–103. doi: 10.5897/AJMR2021.9600

[ref58] Saxena A. K. Kumar M. Chakdar H. Anuroopa N. Bagyaraj D. J. (2020). Bacillus species in soil as a natural resource for plant health and nutrition. J. Appl. Microbiol. 128, 1583–1594. doi: 10.1111/jam.14506, PMID: 31705597

[ref59] Setiawati T. C. Erwin D. Mandala M. Hidayatulah A. (2022). Use of Bacillus as a plant growth-promoting rhizobacteria to improve phosphate and potassium availability in acidic and saline soils. KnE Life Sci. 7, 541–558. doi: 10.18502/kls.v7i3.11160

[ref60] Sharf W. Javaid A. Shoaib A. Khan I. H. (2021). Induction of resistance in chili against Sclerotium rolfsii by plant-growth-promoting rhizobacteria and *Anagallis arvensis*. Egypt J. Biol. Pest. Control. 31, 1–16. doi: 10.1186/s41938-021-00364-y

[ref61] Silva F. C. (2009). Manual de análises químicas de solos, plantas e fertilizantes. Available at: http://www.infoteca.cnptia.embrapa.br/infoteca/handle/doc/330496 (Accessed March 15, 2024).

[ref62] Silva L. I. Pereira M. C. Carvalho A. M. X. Buttrós V. H. Pasqual M. Dória J. (2023). Phosphorus-solubilizing microorganisms: a key to sustainable agriculture. Agriculture 13, 1–30.

[ref63] Singh B. Satyanarayana T. (2011). Microbial phytases in phosphorus acquisition and plant growth promotion. Physiol. Mol. Biol. Plant. 17, 93–103. doi: 10.1007/s12298-011-0062-x, PMID: 23572999 PMC3550544

[ref64] Sousa S. M. Oliveira C. A. Andrade D. L. Carvalho C. G. Ribeiro V. P. Pastina M. M.. (2021). Tropical Bacillus strains inoculation enhances maize root surface area, dry weight, nutrient uptake and grain yield. J. Plant Growth Regul. 40, 867–877. doi: 10.1007/s00344-020-10146-9

[ref65] Stepanović S. Vuković D. Hola V. Di Bonaventura G. Djukić S. Cirković I.. (2007). Quantification of biofilm in microtiter plates: overview of testing conditions and practical recommendations for assessment of biofilm production by staphylococci. APMIS 115, 891–899. doi: 10.1111/j.1600-0463.2007.apm_630.x, PMID: 17696944

[ref66] Tabassum B. Khan A. Tariq M. Ramzan M. Khan M. S. I. Shahid N.. (2017). Bottlenecks in commercialisation and future prospects of PGPR. Appl. Soil Ecol. 121, 102–117. doi: 10.1016/j.apsoil.2017.09.030

[ref67] Tabatabai M. A. (1994). “Soil enzymes” in Methods of soil analysis, part 2. Microbiological and biochemical properties. eds. Weaver R. W. Angle J. S. Bottomley P. S. (Madison, WI: Soil Science Society of America), 775–833.

[ref68] Tabatabai M. A. Bremner J. M. (1969). Use of p-nitrofenol phosphate for assay of soil phosphatase activity. Soil Biol. Biochem. 1, 301–307. doi: 10.1016/0038-0717(69)90012-1, PMID: 38794384

[ref69] Tian J. Ge F. Zhang D. Deng S. Liu X. (2021). Roles of phosphate solubilizing microorganisms from managing soil phosphorus deficiency to mediating biogeochemical P cycle. Biology 10:158. doi: 10.3390/biology10020158, PMID: 33671192 PMC7922199

[ref70] Tisdale S. L. Nelson W. L. (1975). Soil fertility and fertilizers. New York: MacMillan Publishing Co.

[ref71] Vance C. P. Uhde-Stone C. Allan D. L. (2003). Phosphorus acquisition and use: critical adaptations by plants for securing a nonrenewable resource. New Phytol. 157, 423–447. doi: 10.1046/j.1469-8137.2003.00695.x, PMID: 33873400

[ref72] Velloso C. C. Oliveira C. A. Gomes E. A. Lana U. G. P. Carvalho C. G. Guimarães L. J. M.. (2020). Genome-guided insights of tropical Bacillus strains efficient in maize growth promotion. FEMS Microbiol. Ecol. 96:fiaa157. doi: 10.1093/femsec/fiaa157 32785605

