# Correction: Inoculation with *Bacillus megaterium* CNPMS B119 and *Bacillus subtilis* CNPMS B2084 improve P-acquisition and maize yield in Brazil

**DOI:** 10.3389/fmicb.2026.1850844

**Published:** 2026-05-01

**Authors:** Christiane Abreu de Oliveira-Paiva, Daniel Bini, Sylvia Morais de Sousa, Vitória Palhares Ribeiro, Flávia Cristina dos Santos, Ubiraci Gomes de Paula Lana, Fabiane Ferreira de Souza, Eliane Aparecida Gomes, Ivanildo Evódio Marriel

**Affiliations:** 1 Microbiology Laboratory, Embrapa Milho e Sorgo, Sete Lagoas, Brazil; 2 Applied Biology Center, Embrapa Milho e Sorgo, Sete Lagoas, Brazil

**Keywords:** Bacillaceae, bacteria, co-inoculation, maize yield, phosphate-solubilizing, plant growth promoting bacteria

There was a mistake in Table 3 and Table 4 as published. The unit for grain P content was incorrectly reported as g kg^−1^.

**Table 3:** Effects of single inoculation by B119 or B2084 strains on grain P content and maize yield during three seasons (1st, 2nd, and 3rd) at Santo Antônio de Goiás and Sete Lagoas, Brazil.

Treatments	Santo Antônio de Goiás					Sete Lagoas				
	1st	2nd	3rd	Mean	APR (%)^***^	1st	2nd	3rd	Mean	APR (%)
Yield (kg ha^−1^)										
Control^*^	7,615 c	9,413 b	6,648 b	7,892 c	–	2,744 c^**^	1,837 c	3,463 c	2,681 c	–
B0	8,013 c	9,564 b	9,251 a	8,943 b	100	7,178 b	6,751 b	6,910 b	6,886 b	100
B119	10,084 a	9,624 ab	8,878 a	9,529 a	106	8,736 a	9,058 a	7,496 b	8,430 a	122
B2084	8,688 b	10,485 a	8,572 b	9,248 ab	103	8,098 a	6,656 b	9,388 a	8,047 a	116
Grain P content (kg ha^−1^)										
Control	20.2 c	24.4 b	14.1 b	19.5 b	–	5.8 b	2.7 c	7.0 c	5.2 c	–
B0	23.5 b	23.0 b	15.5 ab	20.7 b	100	14.0 a	10.4 b	12.6 b	12.0 b	100
B119	29.0 a	26.8 a	16.0 ab	24.0 a	115	15.7 a	20.1 a	15.2 a	17.3 a	144
B2084	25.1 b	28.4 a	17.1 a	23.5 ab	113	14.3 a	12.2 b	21.0 a	15.9 a	132

^*^Control: no inoculation and zero P fertilizer; B0: no inoculation; B119 (*Bacillus megaterium*); B2084 (*B. subtilis*).

^**^Means followed by different letters in column are significantly different (Duncan *p* ≤ 0.05).

^***^APR: average productivity relative to treatment B0.

**Table 4:** Effects of co-inoculation of B119 and B2084 strains on grain P content and maize yield during two seasons (1st and 2nd) at Santo Antônio de Goiás and Sete Lagoas, Brazil.

Treatments	Santo Antônio de Goiás				Sete Lagoas			
	1st	2nd	Mean	APR (%)^***^	1st	2nd	Mean	APR (%)
Yield (kg ha^−1^)								
Control^*^	8,302 c^**^	7,030 c	7,666 c	–	3,228 c	2,333 c	2,781 c	–
B0	9,413 b	7,970 b	8,692 b	100	8,071 b	5,832 b	6,952 b	100
B119+B2084	10,587 a	8,656 a	9,622 a	111	10,415 a	6,770 a	8,593 a	124
Grain P content (kg ha^−1^)								
Control	31.5 b	22.7 b	34.1 b	–	10.5 c	8.1 b	9.3 c	–
B0	35.7 b	25.7 b	38.7 b	100	26.2 b	20.1 a	23.2 b	100
B119+B2084	45.5 a	38.3 a	43.5 a	112	33.1 a	22.6 a	27.9 a	120

^*^Control: no inoculation and zero P fertilizer; B0: no inoculation; B119 (*Bacillus megaterium*); B2084 (*B. subtilis*).

^**^Means followed by different letters in column are significantly different (Duncan *p* ≤ 0.05).

^***^APR: average productivity relative to treatment B0.

The corrected is kg ha^−1^. The corrected Table 3 and Table 4 appears below.

The original version of this article has been updated.

