# Supplementary material for: Inoculation with Bacillus megaterium CNPMS B119 and Bacillus subtilis CNPMS B2084 improve P-acquisition and maize yield in Brazil
Source: Front Microbiol. 2024 Jun 26;15:1426166. doi: 10.3389/fmicb.2024.1426166 (PMC11233657; doi:10.3389/fmicb.2024.1426166)
Supplement: Supplementary file 1 [file Table_1.DOCX]

Table S1. Soil enzymes activities during two seasons (1^st^ and 2^nd^) at Santo Antônio de Goiás and Sete Lagoas - Brazil after co-inoculation with B119 and B2084 strains.

|  | Acid  phosphatase | Alkaline  phosphatase | | β-  glicosidade | | Arilsulfatase | |  | | | Acid  phosphatase | | | Alkaline  phosphatase | | β-  glicosidade | | Arilsulfatase | |  |  |
| --- | --- | --- | --- | --- | --- | --- | --- | --- | --- | --- | --- | --- | --- | --- | --- | --- | --- | --- | --- | --- | --- |
|  | 1^st^ | | | | | | | | |  | | | 2^nd^ | | | | | | | | |
|  | **Santo Antônio de Goiás** | | | | | | | | | | | | | | | | | | | | |
| Control* | 196.7 b** | | 77.7 b | | 36.9 b | | 67.3 a | |  | | | 128.3 b | | | 61.9 b | | 16.8 b | | 35.6 b | |  |
| B0 | 223.0 ab | | 88.1 b | | 41.8 b | | 76.3 a | |  | | | 145.4 b | | | 70.2 b | | 19.1 b | | 40.3 ab | |  |
| B119+B2084 | 264.9 a | 158.8 a | | 87.5 a | | 83.8 a | |  | | | 226.7 a | | | 107.8 a | | 51.9 a | | 45.4 a | |  |  |
|  | **Sete Lagoas** | | | | | | | | | | | | | | | | | | | | |
| Control | 232.0 c | 53.1 b | | 22.4 b | | 21.7 b | |  | | | 81.4 b | | | 46.5 b | | 48.3 b | | 14.2 b | |  |  |
| B0 | 580.1 b | 132.6 a | | 56.1 a | | 54.4 a | |  | | | 203.6 a | | | 116.3 a | | 120.6 a | | 35.1 a | |  |  |
| B119+B2084 | 627.5 a | 155.1 a | | 52.7 a | | 59.4 a | |  | | | 210.6 a | | | 116.8 a | | 120.8 a | | 32.9 a | |  |  |

* Control: no inoculation and zero P fertilizer; B0: no inoculation; B119 (*Bacillus megaterium*); B2084 (*B. subtilis*).

** Means followed by different letters in column are significantly different (Duncan p ≤ 0.05).
